# A thermostable and alkaline GDSL-motif esterase from *Bacillus* sp. K91: crystallization and X-ray crystallographic analysis

**DOI:** 10.1107/S2053230X18000353

**Published:** 2018-01-26

**Authors:** Junmei Ding, Hujie Zhu, Yujia Ye, Jie Li, Nanyu Han, Qian Wu, Zunxi Huang, Zhaohui Meng

**Affiliations:** a Engineering Research Centre of Sustainable Development and Utilization of Biomass Energy, Ministry of Education, Yunnan Normal University, 768 Juxian Road, Kunming, Yunnan 650500, People’s Republic of China; b Laboratory of Molecular Cardiology, Department of Cardiology, The First Affiliated Hospital of Kunming Medical University, 1168 Chunrong West Road, Kunming, Yunnan 650500, People’s Republic of China; c Fudan University Shanghai Centre, Institute of Biomedical Science, Fudan University, 131 Dong’an Road, Shanghai 200032, People’s Republic of China

**Keywords:** Est8, crystallization, X-ray crystallographic analysis, GDSL-motif esterase

## Abstract

The esterase Est8 from the thermophilic bacterium *Bacillus* sp. K91 belongs to the GDSL family and is active on a variety of acetylated compounds, including 7-aminocephalosporanic acid. In contrast to other esterases of the GDSL family, the catalytic residues Asp182 and His185 were more pivotal for the catalytic activity of Est8 than the Ser11 residue. To better understand the biochemical and enzymatic properties of Est8, recombinant Est8 protein was purified and crystallized. Crystals of Est8 were obtained by the hanging-drop vapour-diffusion method using 2.0 *M* ammonium sulfate, 5%(*v*/*v*) 2-propanol as the crystallization solution. X-ray diffraction data were collected to a resolution of 2.30 Å with an *R*_merge_ of 16.4% from a crystal belonging to space group *P*4_1_2_1_2 or *P*4_3_2_1_2, with unit-cell parameters *a* = *b* = 68.50, *c* = 79.57 Å.

## Abstract 2

Purification, crystallization and X-ray crystallographic analysis were employed to determine the catalytic mechanism of Est8, a GDSL-motif esterase from *Bacillus* sp. K91.

## 1. Introduction   

The esterases (EC 3.1.1.1) and lipases (EC 3.1.1.3) are collectively known as lipolytic enzymes, and have important physiological and biotechnological roles in the synthesis or hydrolysis of ester-containing compounds (Rubin, 1994 ▸). Although there are similarities in their molecular structures and catalytic mechanisms, esterases can be distinguished from true lipases by their preference for fatty-acid esters with acyl-chain lengths of less than ten C atoms, whereas lipases display maximal activity towards water-insoluble long-chain tri­glycerides (Jaeger & Eggert, 2002 ▸). Common esterases possess the highly conserved motif Gly-*X*-Ser-*X*-Gly, which contains the catalytic Ser residue (López-López *et al.*, 2014 ▸; Stergiou *et al.*, 2013 ▸).

As important biocatalysts, lipolytic enzymes are currently attracting enormous attention owing to their numerous applications in industry, such as in the food, leather, fine chemical, detergent, cosmetics, pharmaceutical and paper industries. Although lipolytic enzymes exist in various species, the most widely exploited enzymes are produced by microorganisms (Bornscheuer, 2002 ▸). Among them, thermostable lipolytic enzymes are always in great demand because most industrial lipolytic hydrolase and esterification reactions take place under harsh conditions such as at high temperature or under organic solvent conditions. They are better suited to the harsh processes involved in industrial and biotechnological applications (Hess *et al.*, 2008 ▸; Hotta *et al.*, 2002 ▸).

**Figure 1:**
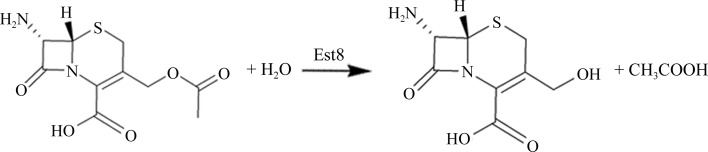
Reaction scheme for the esterase using 7-ACA as a substrate.

Recently, we have cloned and characterized a thermostable esterase gene *est8* from *Bacillus* sp. K91 (accession No. KJ131182; Ding *et al.*, 2014 ▸). A comparison of its amino-acid sequence showed that Est8 could be grouped into the GDSL family. The GDSL-like (Pfam PF00657) subfamily is characterized by the presence of a distinct GDSL motif that differs from the classical G*X*S*X*G motif found in many lipolytic enzymes (Akoh *et al.*, 2004 ▸). Est8 can be further classified as a member of the SGNH hydrolase superfamily because of the presence of four strictly conserved and functionally important residues, Ser, Gly, Asn and His, in conserved blocks I, II, III and V, respectively (Mølgaard *et al.*, 2000 ▸). Recombinant Est8 was identified as an esterase which prefers to hydrolyze short *p*-nitrophenyl esters of acetate (C_2_). Est8 displayed maximum activity around 323 K at pH 9.0. Interestingly, it also retained relatively high activity in low- and high-temperature environ­ments, displaying 40 and 20% of its maximum activity at 293 and 368 K, respectively (Ding *et al.*, 2014 ▸). Est8 might be of significant industrial interest and value in scientific research as a thermostable esterase, and exhibits maximum activity in an alkaline environment. Moreover, Est8 can hydrolyze acetate at the C3 position of 7-aminocephalo­sporanic acid (7-ACA) to form deacetyl-7-ACA (D-7-ACA), as shown in Fig. 1 ▸ (data not shown). D-7-ACA is an important starting material for the production of semisynthetic β-lactam antibiotics (Levisson *et al.*, 2012 ▸; Tian *et al.*, 2014 ▸). To provide new insights into the structure–function relationship of Est8 and to obtain a better understanding of the biochemical data, determination of the crystal structure of the enzyme is in progress.

## 2. Materials and methods   

### 2.1. Macromolecule production   

The cloning, expression and purification of Est8, with the exception of gel filtration, have been described previously by Ding *et al.* (2014 ▸). Here, we describe the procedure in more detail. The gene encoding the enzyme was PCR-amplified from the genomic DNA of the thermophilic bacterium *Bacillus* sp. K91 using the primers 5′-GCAAATCATATTTATCTTGC-3′ (N-terminal) and 5′-CCTTTCTTTGATGATCGATTC-3′ (C-terminal). The PCR product was subsequently directly ligated with the pEASY-E2 expression vector according to the manufacturer’s instructions. A hexahistidine tag (LEHHHHHH) was added from the vector sequence at the C-terminal end of the recombinant protein (Table 1 ▸). The ligation mixture was then transformed into *Escherichia coli* BL21 (DE3) cells (Novagen). Individual constructs were isolated from the transformants, screened for the correct size insert and completely sequenced to confirm their nucleotide identity. The transformed strains were grown in LB medium containing 100 mg ml^−1^ ampicillin at 310 K until the OD_600_ reached 0.6. Following induction with 0.7 m*M* isopropyl β-d-1-thiogalactopyranoside (IPTG) at 293 K for 20 h, the cells were harvested by centrifugation and disrupted by sonication. The lysate was centrifuged at 15 000*g* for 30 min and the supernatant was applied onto an Ni^2+^–NTA agarose-gel column for purification using a linear imidazole gradient of 20–500 m*M* in buffer *A* (20 m*M* Tris–HCl, 0.5 *M* NaCl pH 7.2). The target protein was eluted with buffer *A* containing 80 m*M* imidazole. The obtained Est8 protein was further purified by gel filtration using a Superdex 75 10/300 GL column (GE Healthcare) in running buffer consisting of 20 m*M* Tris–HCl, 0.5 *M* NaCl pH 7.2. Finally, the pure Est8 protein was concentrated to 10–15 mg ml^−1^ using an Amicon Ultra-15 centrifugal filter unit (10 000 nominal molecular-weight limit; Millipore) for crystallization.

### 2.2. Crystallization   

Initial crystallization of Est8 was attempted by the hanging-drop vapour-diffusion method at 289 K using the Crystal Screen, Crystal Screen 2, PEG/Ion and SaltRx reagent kits (Hampton Research). 1 ml protein solution was mixed with 1 ml reservoir solution and the mixture was equilibrated against 200 ml reservoir solution. Fusiform-shaped crystals were observed after 4 d using buffer consisting of 2.0 *M* ammonium sulfate, 5%(*v*/*v*) 2-propanol. The crystallization conditions were optimized *via* variation of the ammonium sulfate concentration (0.5–5.0 *M*), 2-propanol concentration [2–8%(*v*/*v*)] and protein concentration (to a maximum of 15 mg ml^−1^) at 289 K in 16-well plates (Table 2 ▸).

### 2.3. Data collection and processing   

Before data collection, the crystals were soaked in mother liquor containing 20%(*v*/*v*) glycerol as a cryoprotectant and were flash-cooled directly in liquid nitrogen. Data sets were collected on beamline BL17U1 at the Shanghai Synchrotron Radiation Facility (SSRF), People’s Republic of China using an ADSC Q315 CCD system (Wang *et al.*, 2015 ▸). The exposure time was 1.0 s per frame. One complete data set was obtained by collecting 180 frames with 1° oscillation. The data collected were processed and scaled with the *HKL*-2000 suite (Otwinowski & Minor, 1997 ▸).

## 3. Results and discussion   

**Figure 2:**
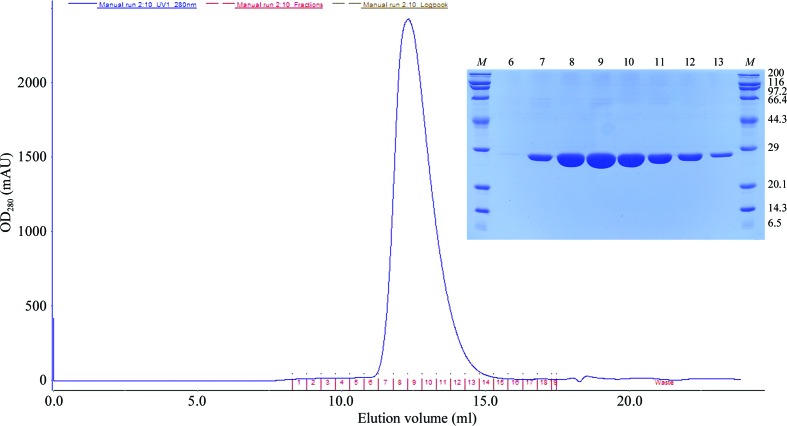
Size-exclusion chromatography and SDS–PAGE (inset) of Est8. In the SDS–PAGE, lane *M* contains molecular-weight markers (labelled in kDa) and lanes 6–13 contain purified Est8 obtained at different volumes of elution buffer as shown on the size-exclusion chromatogram.

Est8 from *Bacillus* sp. K91, with a calculated molecular mass of 24.5 kDa and a theoretical isoelectric point of 6.45, was cloned to contain a C-terminal 6×His tag, overproduced in *E. coli* BL21 (DE3) cells and purified to homogeneity in a two-step procedure by Ni–NTA affinity and size-exclusion chromatography for crystallization (Fig. 2 ▸). The final purity of Est8 was at least 99% as estimated by SDS–PAGE, and gel filtration indicated that it was monomeric. Significant enzyme activity was maintained after purification (Ding *et al.*, 2014 ▸).

**Figure 3:**
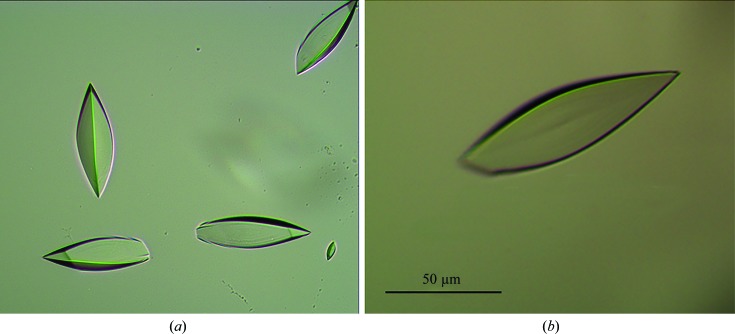
Pictures of Est8 protein crystals. (*a*) Initial crystallization screening with Est8 in 20 m*M* Tris–HCl, 0.5 *M* NaCl pH 7.2 using the crystallization condition 2.0 *M* ammonium sulfate, 5%(*v*/*v*) 2-propanol. (*b*) Picture of an Est8 crystal.

**Figure 4:**
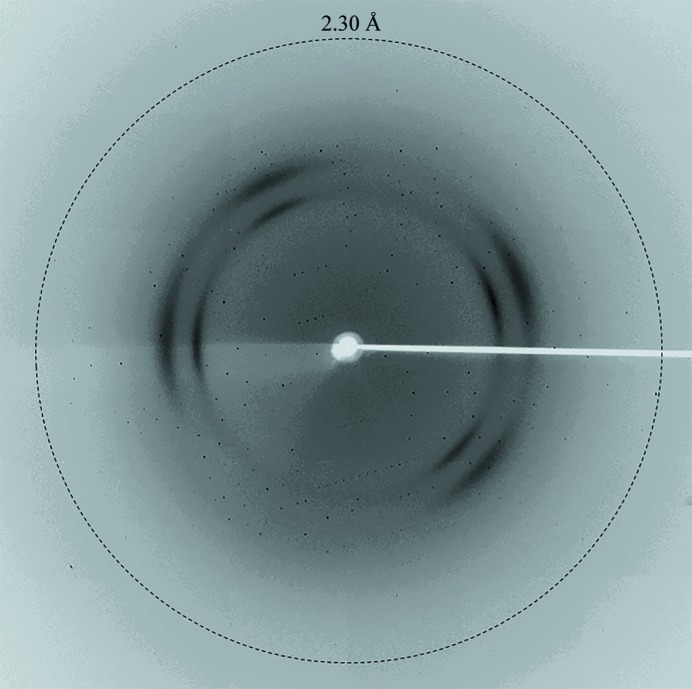
A typical X-ray diffraction pattern collected from an Est8 protein crystal. The diffraction image was collected on beamline BL17U1 at SSRF using an ADSC Q315 CCD detector. The detector edge corresponds to ∼2.0 Å resolution.

Purified recombinant Est8 enzyme, which can be stored at 277 K for several weeks without suffering protein degradation, was concentrated to 10–15 mg ml^−1^ before being subjected to crystallization. Initial crystallization screening showed that a reservoir solution consisting of 2.0 *M* ammonium sulfate, 5%(*v*/*v*) 2-propanol could be used to grow Est8 crystals. Attempts were made to optimize the crystallization conditions for Est8 by varying the concentration of ammonium sulfate (0.5–5.0 *M*) and 2-propanol [4–7.5%(*v*/*v*)] based on the initial crystallization conditions. Crystals could be grown under conditions consisting of 2.0 *M* ammonium sulfate with 2-propanol at a concentration ranging from 4 to 7.5%(*v*/*v*). Thus, sufficiently large crystals were obtained both with the initial condition from the kit and using optimized conditions (Fig. 3 ▸ *a*). The dimensions of the crystals were approximately 0.05 × 0.05 × 0.1 mm (Fig. 3 ▸ *b*). The crystals diffracted to 2.30 Å resolution and belonged to space group *P*4_1_2_1_2 or *P*4_3_2_1_2, with unit-cell parameters *a* = *b* = 68.50, *c* = 79.57 Å (Fig. 4 ▸). Assuming the presence of one molecule in the asymmetric unit, the Matthews coefficient *V*_M_ was calculated to be 1.90 Å ^3^ Da^−1^, with a solvent content of approximately 35.2%. The statistics of data collection are summarized in Table 3 ▸. Initial phase determination was carried out by molecular replacement with *Phaser* using the crystal structure of the hypothetical YxiM precursor from *B. subtilis* (PDB entry 2o14, chain *A*; Northeast Structural Genomics Consortium, unpublished work) as a search model. This protein belongs to the SGNH hydrolase family, as does Est8, and displayed 35% identity to Est8. Owing to the low amino-acid sequence identity with Est8, attempts to solve the structure of Est8 using molecular replacement failed. Therefore, the preparation of selenomethionyl-derivatized Est8 protein and heavy-atom soaking is currently under way for the structure determination of the GDSL-family thermostable esterase Est8. Once structural information for Est8 has been obtained, it will be used as a starting point to better understand its catalytic mechanism and potential applications in industry.

## Figures and Tables

**Table 1:** Macromolecule-production information

Source organism	*Bacillus* sp. K91
DNA source	*Bacillus* sp. K91
Forward primer	5′-GCAAATCATATTTATCTTGC-3′
Reverse primer	5′-CCTTTCTTTGATGATCGATTC-3′
Expression vector	pEASY-E2
Expression host	*E. coli* BL21 (DE3)
Complete amino-acid sequence of the construct produced	MANHIYLAGDSTVQTYGDSTNQGGWGQFLGSHLPEHIQVINRAIGGRSSKTFVEEGRLQAILDVIEPDDWLFVQMGHNDASKNKPERYTEPYTTYKQYLKQYIAGAREKGAHPLLITPVARFHYENGVFLNDFPDYCIAMKQTAEEENVQLIDLMEKSLAFFTEKGEEKVYTYFMISEGINDYTHFTKKGANEMAKLVAKGIKELGLPLTESIIKERLEHHHHHH

**Table 2:** Crystallization

Method	Hanging-drop vapour diffusion
Plate type	16-well Linbro cell-culture plates
Temperature (K)	289
Protein concentration	12
Buffer composition of protein solution	20 m*M* Tris–HCl, 0.5 *M* NaCl pH 7.2
Composition of reservoir solution	2.0 *M* ammonium sulfate, 5%(*v*/*v*) 2-propanol
Volume and ratio of drop	2 µl, 1:1
Volume of reservoir (µl)	200

**Table 3:** Data collection and processing

Diffraction source	Beamline BL17U1, SSRF
Wavelength (Å)	0.9792
Temperature (K)	100
Detector	ADSC Q315 CCD
Crystal-to-detector distance (mm)	300
Rotation range per image (°)	1
Total rotation range (°)	180
Exposure time per image (s)	1
Space group	*P*4_1_2_1_2 or *P*4_3_2_1_2
Unit-cell parameters (Å)	*a* = *b* = 68.50, *c* = 79.57
Resolution range (Å)	50–2.30 (2.38–2.30)
Total No. of reflections	54346
No. of unique reflections	8899 (858)
Completeness (%)	100 (100)
Multiplicity	6.1 (6.3)
*R*_merge_ † (%)	16.4 (60.5)
*V*_M_ (Å^3^ Da^−1^)	1.90
Mean *I*/σ(*I*)	14.8 (3.3)
Overall *B* factor from Wilson plot (Å^2^)	15.9

† *R*_merge_ = 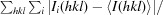 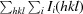, where 〈*I*(*hkl*)〉 is the mean intensity of the observations *I_i_*(*hkl*) of reflection *hkl*.
